# Nutrition Provision in Pediatric Extracorporeal Membrane Oxygenation: Evidence, Challenges, and Clinical Considerations

**DOI:** 10.3390/nu17091553

**Published:** 2025-04-30

**Authors:** Marwa Mansour, Nicole Knebusch, Andrea Ontaneda, Stephanie Vazquez, Jennifer Daughtry, Katri Typpo, Jorge A. Coss-Bu

**Affiliations:** 1 Division of Critical Care Medicine, Department of Pediatrics, Baylor College of Medicine, Houston, TX 77030, USA; 2 Texas Children’s Hospital, Houston, TX 77030, USA; 3 Department of Clinical Nutrition Services, Texas Children’s Hospital, Houston, TX 77030, USA

**Keywords:** pediatric ECMO, nutrition support, enteral nutrition, parenteral nutrition, feeding intolerance, critical care nutrition, malnutrition, extracorporeal therapy

## Abstract

Background/Objectives: Nutritional support is a critical yet challenging aspect of care for pediatric patients requiring extracorporeal membrane oxygenation (ECMO). Malnutrition is prevalent in this population and is associated with worse clinical outcomes. This review synthesizes current evidence on nutritional strategies for pediatric ECMO patients, emphasizing assessment methods, feeding routes, challenges, and clinical outcomes. Methods: A literature review was conducted using PubMed, Scopus, and Web of Science to identify relevant studies published between January 2010 and 2025. Keywords included “pediatric ECMO”, “nutrition”, “enteral feeding”, and “parenteral nutrition”. Studies addressing nutritional assessment, enteral and parenteral feeding practices, and their impact on clinical outcomes were included. Results: Malnutrition is a significant risk factor for morbidity and mortality in ECMO patients, yet nutritional support remains highly variable. While enteral nutrition (EN) is preferred, feeding intolerance and gastrointestinal dysfunction often necessitate parenteral nutrition (PN). Early EN initiation, even at trophic levels, is associated with improved gut integrity and outcomes. However, achieving full nutritional goals enterally remains a challenge, particularly in neonates. PN remains essential in cases of feeding intolerance but is linked to hepatic dysfunction and metabolic imbalances. Conclusions: Optimizing nutritional support in pediatric ECMO patients requires individualized assessment and a structured approach to enteral and parenteral feeding. Further research is needed to establish standardized feeding protocols and determine the optimal timing and composition of nutritional support to improve outcomes.

## 1. Introduction

Extracorporeal membrane oxygenation (ECMO) is a life-saving therapy used in pediatric intensive care units (PICUs) for critically ill infants and children, providing temporary respiratory and/or circulatory support in conditions such as severe respiratory failure, cardiac arrest, or shock [1,2]. As the use of ECMO has expanded in pediatric care, particularly for complex cases, it is essential to address the unique nutritional challenges these patients face [2,3].

Children on ECMO encounter significant nutritional challenges due to the underlying conditions that necessitate ECMO support, the physiological effects of ECMO itself, the systemic inflammatory response, coupled with sedation, and immobility, increasing energy expenditure and protein breakdown [4,5]. Additionally, feeding intolerance resulting from gastrointestinal dysmotility, hemodynamic instability, or fluid restrictions further complicates the provision of adequate nutrition in these patients. Prolonged periods of suboptimal nutrition have been associated with increased morbidity, prolonged ECMO duration, and poor long-term outcomes, underscoring the critical importance of optimizing nutritional delivery in this population [4,6]. Undernutrition is prevalent in this population, and such deficits can worsen prognosis and hinder recovery [7]. Thus, optimizing nutritional support is critical to improving outcomes in pediatric ECMO patients.

Despite the recognized importance of nutrition in critically ill children, there remains considerable uncertainty regarding the optimal approach to nutritional support in pediatric ECMO patients. Several unanswered questions persist, including the appropriate timing for feeding initiation, the optimal route of nutrition (enteral vs. parenteral), target caloric and protein delivery, and strategies to overcome feeding intolerance in this high-risk population [6,8,9]. Current clinical practices are largely guided by expert consensus, extrapolated data from non-ECMO critically ill populations, or small observational studies, highlighting the urgent need for high-quality research to inform evidence-based guidelines for this vulnerable population [10,11].

This review synthesizes current evidence on nutritional strategies for pediatric ECMO patients, focusing on nutritional assessment, feeding routes (enteral vs. parenteral), delivery challenges, and the impact of nutritional interventions on clinical outcomes. We will critically evaluate the existing literature, highlighting areas of consensus and controversy, and identify gaps that warrant further investigation.

## 2. Materials and Methods

We conducted a comprehensive literature search across multiple medical databases, including PubMed, Embase, Scopus, and Web of Science. The search focused on articles published between January 2010 and 2024, prioritizing studies that examined nutritional requirements, feeding strategies, and outcomes in critically ill children on extracorporeal support. The search strategy used a combination of Medical Subject Headings (MeSH) and free-text keywords such as “pediatric ECMO”, “extracorporeal membrane oxygenation”, “enteral nutrition”, “parenteral nutrition”, “feeding tolerance”, and “nutritional adequacy”. The final selection included original research articles providing quantitative data on nutrition delivery, adequacy, and clinical outcomes in pediatric ECMO patients (Table 1).

## 3. Discussion

### 3.1. ECMO Use in PICU and NICU: Uses and Trends

ECMO is a life-support technology used in patients with cardiac and/or respiratory failure refractory to conventional medical management. There are two major types of ECMO: veno-venous (VV) ECMO, which provides respiratory support by removing blood from the venous system, passing it through an oxygenator, and then returning the oxygenated blood into the venous circulation via the right atrium; and veno-arterial (VA) ECMO, which provides cardiac as well as respiratory support by directly returning oxygenated blood into the arterial system [22]. In children and neonates, ECMO could be used to manage respiratory failure, congenital diaphragmatic hernia, sepsis, and cardiac failure due to congenital heart defects or myocarditis, among other conditions [23].

The most recent Extracorporeal Life Support Organization (ELSO) Registry International Summary data up to 2023 help us understand the current trends in ECMO and the use of ECMO for critically ill pediatric and neonatal patients. ECMO support has grown significantly over the years, both in availability and use. ECMO support has increased markedly over time in terms of availability and use. In 1990, only 83 centers provided ECMO, but by 2023, that number had increased to 593, with a significant increase in ECMO runs. These numbers show that ECMO has become a critical tool in saving lives, though there is still work to be done. While pulmonary ECMO offers the best outcomes, cardiac ECMO and extracorporeal cardiopulmonary resuscitation (ECPR) remain more complex, highlighting the need for continued advancements in patient care, technology, and management strategies to improve survival rates, especially for the sickest patients.

In neonates, pulmonary ECMO has high survival rates, with 72% surviving to discharge or transfer. Cardiac ECMO for neonates, however, has a lower survival rate of 45%, and neonatal ECPR, often used in emergency resuscitation, has an overall survival rate of 42%. Pulmonary ECMO for pediatric patients has a survival rate of 62%, while cardiac ECMO survival is slightly lower at 55%. Pediatric ECPR remains the most challenging, with only a 41% survival rate [24]. Furthermore, associated complications such as bleeding and infection have been fewer due to better anticoagulation and prevention protocols.

Nutrition management in ECMO patients is challenging due to their complex physiologic and hemodynamic status. Patients on ECMO who experience changes in blood flow and blood pressure, especially in the setting of splanchnic perfusion compromise, are at high risk of developing gastrointestinal (GI) function abnormalities, leading to reductions in nutrient absorption. ECMO patients with hemodynamic instability have problems with nutrient digestion and absorption. Enteral nutrition, usually the preferred route, may be poorly tolerated due to these physiologic changes while on ECMO support. Furthermore, anticoagulation therapy on ECMO increases the risk of GI bleeding, which complicates the enteral feeding process [4,25].

### 3.2. Metabolic Alterations and Nutritional Demands in Pediatric ECMO Patients

Critically ill children receiving ECMO experience profound metabolic alterations, which can manifest as either hypermetabolism or hypometabolism, depending on the severity of illness and underlying condition [1,2]. Contrary to the concept of metabolic rest, ECMO does not attenuate catabolism. Patients on ECMO exhibit some of the highest rates of protein breakdown reported in critically ill populations [26,27,28].

A systemic inflammatory response frequently drives a hypermetabolic state in these patients, leading to increased energy demands that are challenging to meet due to feeding intolerance, protein catabolism, and impaired nutrient absorption [25]. Enteral feeding intolerance is common and often results from intestinal hypoperfusion, gut edema, and delayed gastric emptying, necessitating parenteral nutrition in some cases [15,16]. While parenteral nutrition can temporarily meet metabolic needs, its use is associated with significant risks, including liver dysfunction and infection [29].

Beyond these challenges, ECMO itself contributes to nutritional deficits. Hemolysis and circuit-related losses increase protein and micronutrient requirements, while anticoagulation therapy may exacerbate micronutrient deficiencies, such as vitamin K depletion. Additionally, renal dysfunction, common in ECMO patients, can lead to electrolyte imbalances that further compromise nutritional status [29,30]. Given these factors, an increased protein intake is necessary to counteract muscle wasting and support tissue repair [26]. However, energy requirements vary widely, influenced by patient age, underlying disease, and illness severity, making precise estimation challenging. In practice, nutritional goals often rely on extrapolated data from other critically ill populations in the absence of standardized ECMO-specific guidelines [1,2,3].

### 3.3. Malnutrition and Outcomes in Pediatric ECMO

Malnutrition is highly prevalent among critically ill pediatric patients and is strongly associated with worse clinical outcomes [31,32]. Current guidelines emphasize the importance of early nutritional assessment upon admission to identify malnourished patients or those at risk, allowing for timely interventions to optimize nutritional support [10,26].

In pediatric patients receiving ECMO, underweight status is an independent predictor of in-hospital mortality [7]. Many of these patients are already critically ill before ECMO initiation, placing them at heightened risk for malnutrition. The combination of severe illness and suboptimal nutritional status has been associated with prolonged ECMO duration and adverse clinical outcomes. Furthermore, delivering adequate nutrition during ECMO remains challenging due to feeding intolerance, metabolic derangements, and the severity of critical illness [3,33].

Malnutrition is a significant risk factor for increased morbidity and mortality in pediatric ECMO patients. Undernourished patients face higher rates of infection, multiple organ dysfunction syndrome, and prolonged mechanical ventilation [3,7,30]. The systemic inflammatory response characteristic of critical illness further exacerbates metabolic demands while simultaneously impairing nutrient absorption and utilization. Studies evaluating nutritional support in ECMO patients have demonstrated that inadequate caloric intake and delays in feeding initiation are associated with increased mortality, underscoring the importance of early enteral feeding and optimized nutritional support in improving survival and long-term outcomes [1,6,21,34].

Among ECMO survivors, malnutrition significantly impacts post-ECMO recovery. Poor nutritional status not only affects short-term outcomes but also contributes to long-term complications. Many survivors require extensive rehabilitation due to muscle atrophy, which is exacerbated by prolonged sedation, neuromuscular blockade, and inadequate protein intake [25,35]. In neonates, malnutrition during ECMO has profound implications for growth and neurodevelopment, with studies indicating an increased risk of cognitive and motor deficits among those who experienced nutritional deficiencies in the neonatal period [1]. Given these potential long-term consequences, ensuring optimal nutrition during ECMO is particularly crucial in neonatal populations.

Malnutrition remains a critical challenge in pediatric and neonatal ECMO patients, significantly influencing morbidity, mortality, and long-term outcomes. Early nutritional assessment, timely initiation of enteral feeding, and careful monitoring of energy and protein demands are essential components of ECMO management. By prioritizing optimized nutritional strategies, clinicians can improve survival, facilitate recovery, and enhance the long-term quality of life for this vulnerable population [7,30,31].

### 3.4. Nutritional Assessment While on ECMO

An accurate assessment of nutritional status is essential for guiding individualized nutrition strategies in pediatric ECMO patients. However, the heterogeneity of this population, with varying underlying pathologies and ECMO durations, presents significant challenges [20]. While established methods exist for assessing nutritional status in critically ill children, their applicability to the unique context of ECMO requires further investigation [2].

Regular monitoring of weight, length/height, and anthropometric measurements remains a fundamental component of nutritional assessment, complemented by biochemical markers such as albumin and prealbumin levels [1]. However, these traditional measures may not fully capture the metabolic alterations associated with ECMO.

Indirect calorimetry (IC) is considered the gold standard for measuring resting energy expenditure (REE) and provides a more accurate estimation of energy needs compared to predictive equations [32,36]. However, its widespread use in pediatric critical care is limited due to accessibility constraints and technical challenges, particularly in ECMO patients [2,32,37]. Patients on ECMO, whose blood is oxygenated externally by the circuit, lack the physiological conditions required for accurate IC measurements, further complicating its application [38].

A study evaluating IC in six pediatric ECMO patients attempted to circumvent these challenges by connecting an indirect calorimeter to the mechanical ventilator and analyzing blood gases obtained directly from the ECMO circuit to determine gas exchange and REE [12]. The results demonstrated significant variability in REE values compared to predictive estimates on day 2 of ECMO support, ranging from 26 to 154 kcal/kg/day. Notably, patients with septic shock exhibited an REE increase exceeding 300% of predicted values [12]. While this study highlights the limitations of predictive equations, it also underscores the difficulty in validating IC accuracy under these conditions.

Similarly, a study by Li et al. utilized respiratory mass spectrometry to evaluate energy expenditure (EE) and respiratory quotient (RQ) in five children on ECMO. In patients aged 0.3 to 36 months, EE was found to range between 40 and 46 kcal/kg/day lower than anticipated for a hypermetabolic, hypercatabolic state [3,39]. These findings further illustrate the complexity of REE estimation in ECMO patients and highlight the influence of underlying pathology and pre-ECMO nutritional status [39].

Given the limitations of IC in ECMO patients, the American Society for Parenteral and Enteral Nutrition (ASPEN) recommends using Schofield’s equation to estimate basal metabolic needs when direct measurements are unavailable [26,32,40]. Current guidelines also advocate for a minimum protein intake of 1.5 g/kg/day to maintain a positive nitrogen balance and support metabolic demands [32,41].

Additionally, the impact of continuous renal replacement therapy (CRRT), frequently employed in ECMO patients, must be carefully considered, as it can alter nutritional requirements and contribute to significant micronutrient losses [1,28]. The absence of standardized assessment tools and protocols further underscores the need for ongoing research to refine nutritional assessment and optimize nutrition delivery in this high-risk population [3].

### 3.5. Nutritional Support While on ECMO: Quantity and Route

Nutritional support refers to the provision of essential macronutrients and micronutrients to critically ill patients to maintain metabolic homeostasis, preserve lean body mass, and promote recovery. In the context of pediatric and neonatal ECMO patients, nutritional support is vital for mitigating the risks of malnutrition, supporting immune function, and optimizing clinical outcomes [35].

Both underfeeding and overfeeding carry significant consequences. Underfeeding is associated with impaired wound healing, myocardial mass loss, and increased mortality, whereas excessive nutrition can contribute to hepatic steatosis and liver dysfunction [3]. Optimizing nutrition is therefore crucial to improving outcomes in this vulnerable population [1,2]. A recent survey of pediatric ECMO centers in the US and Canada revealed considerable variability in nutritional practices, underscoring the need for further research and the development of standardized protocols [20].

Providing nutritional support to children on ECMO presents numerous challenges, primarily due to uncertain metabolic demands and the vulnerable physiological state of these patients. Critically ill children on ECMO are often in a hypermetabolic and hypercatabolic state, necessitating precise nutritional management to meet their increased energy and protein requirements. However, multiple factors, including the use of sedatives, concurrent medications, frequent procedures, and fluid restrictions, complicate the ability to achieve optimal nutritional goals. These constraints make both the calculation of nutritional requirements and their successful delivery particularly difficult [11,32].

**Figure 1:**
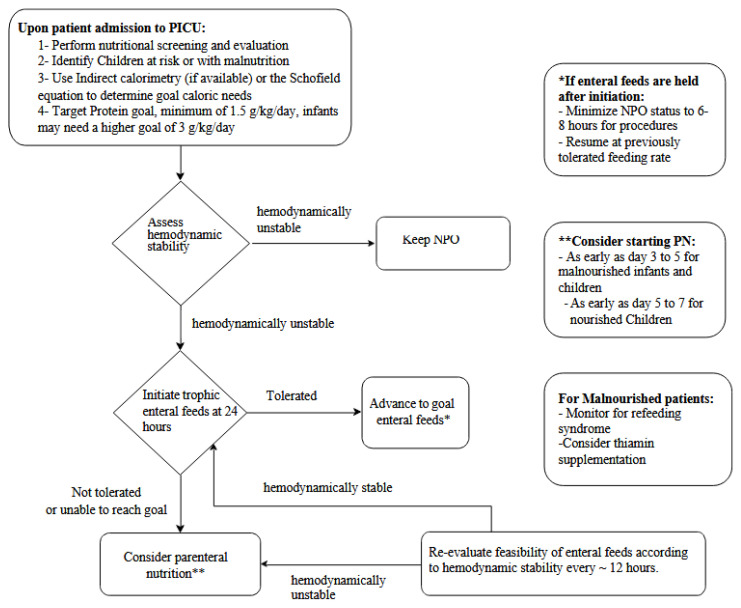
Nutrition support pathway for critically ill children in a PICU (adapted from Texas Children’s Hospital nutrition provision protocol) [42].

The selection of an appropriate nutrition route, whether enteral nutrition (EN) or parenteral nutrition (PN), depends on the patient’s clinical stability, underlying condition, and institutional protocols (Figure 1) [42]. While EN is generally preferred due to its benefits in maintaining gut integrity and reducing infection risks, PN is often necessary when enteral feeding is not feasible due to gastrointestinal dysfunction or hemodynamic instability.

Despite the importance of nutrition in ECMO patients, there remains a lack of high-quality evidence regarding the optimal amount, route, and timing of nutritional support, with few randomized controlled trials (RCTs) available. Nonetheless, consensus guidelines from major nutrition societies, including the American Society for Parenteral and Enteral Nutrition (ASPEN) and the European Society of Clinical Nutrition and Metabolism (ESPEN), recommend enteral nutrition as the preferred route of nutrient delivery whenever feasible [3,15,32,43]. These recommendations emphasize the importance of early enteral feeding initiation to improve outcomes, provided the patient’s clinical status allows for safe gastrointestinal function.

### 3.6. Enteral Nutrition in Pediatric ECMO: Benefits and Challenges

Enteral nutrition (EN), the delivery of nutrients through the gastrointestinal (GI) tract, is generally considered the preferred route of nutritional support for pediatric ECMO patients due to its well-documented benefits in maintaining gut integrity, reducing infection risks, and preserving gut-associated lymphoid tissue (GALT) function [1,2,3]. Additionally, EN helps sustain the microbiome, reducing bacterial translocation and systemic inflammation [34].

Early initiation of EN, ideally within 24–48 h of ECMO initiation, has been associated with improved outcomes, including reduced mortality and enhanced gut barrier function [1,2,3,44]. However, concerns about GI complications such as splanchnic hypoperfusion, bowel ischemia, and perforation have historically led to hesitancy in initiating EN. Despite these concerns, multiple studies have demonstrated that EN does not increase GI complications in ECMO patients [6,13,16,17,18]. Research across both veno-venous (VV) and veno-arterial (VA) ECMO configurations, including patients on non-pulsatile flow, has further confirmed its safety and feasibility [8,19].

While EN is considered safe, parenteral nutrition (PN) continues to be widely used, either alone or as an adjunct to trophic EN, due to challenges in meeting full nutritional goals enterally. Many studies suggest that early initiation of trophic EN, with gradual advancement as tolerated, is not associated with increased complications [6,13,14,17,18,19,39].

A systematic review and meta-analysis of 14 studies involving 1650 pediatric ECMO patients found that EN was not associated with increased harm compared to PN. No significant differences were observed in sepsis rates, intestinal bacterial infections, or major abdominal complications (such as bowel perforation and necrotizing enterocolitis) between enterally fed and parenterally fed patients. Notably, EN was deemed safe even in neonatal ECMO patients, with no significant increase in abdominal complications compared to PN [39].

### 3.7. Challenges and Barriers to Enteral Nutrition

Despite its advantages, advancing enteral feeds remains challenging due to various patient-related and ECMO-specific factors. The most frequently reported issue is feeding intolerance, often manifested as vomiting, gastric retention, and inadequate nutrient delivery (Table 2) [1,39]. Certain patient populations, including neonates, those on VA-ECMO, and patients with congenital diaphragmatic hernia (CDH), are at a higher risk of feeding intolerance and are less likely to receive EN [16].

Delayed gastric emptying and bowel ischemia further complicate feeding tolerance in ECMO patients [2]. However, the use of minimal enteral nutrition (MEN) as trophic feeds has been shown to stimulate gut motility, preserve intestinal integrity, and improve tolerance without increasing complications [5].

Optimizing EN delivery remains a crucial goal, as achieving caloric and protein targets via EN alone can be challenging due to hemodynamic instability and fluid restrictions. Studies suggest that a hybrid approach, combining early low-dose EN with PN, may help prevent cumulative protein deficits while ensuring adequate caloric intake [16].

### 3.8. Enteral Nutrition and Survival Outcomes

Several studies indicate a positive correlation between EN adequacy and survival in pediatric ECMO patients. A study by Chengsi Ong et al. found that greater EN adequacy was associated with lower mortality rates, reinforcing the need for optimized enteral feeding strategies [15].

Similarly, a study of 24 pediatric ECMO patients demonstrated that those who received EN had a significantly higher survival rate at day 5 compared to those who received PN alone (*p* = 0.031). However, this difference was no longer significant beyond day 5, suggesting that the early phase of ECMO support may be a critical window for nutrition optimization [6].

A larger cohort study of 51 children on ECMO found that survivors received significantly more enteral nutrition within the first 7 days post-ECMO initiation compared to non-survivors. After adjusting for renal replacement therapy and vasoactive medication use, the relationship remained statistically significant (adjusted OR: 0.93 [95% CI: 0.86–0.99], *p* = 0.048) [15]. These findings suggest that early enteral nutrition within the first week of ECMO is not only feasible but also associated with improved survival.

The recent evidence supports the feasibility and safety of early EN in hemodynamically stable ECMO patients. For instance, Dennis et al. demonstrated that early EN was associated with better survival outcomes and reduced complications compared to delayed EN [44]. Similarly, Li et al. found that initiating EN within 48–72 h of ECMO initiation correlated with improved clinical outcomes and reduced hospital LOS [50].

### 3.9. Parenteral Nutrition (PN)

Parenteral nutrition (PN) serves as a supplementary or primary method of nutrition support when EN is insufficient or contraindicated [1,2,14,16]. While PN can provide adequate caloric and protein intake, it is associated with various complications, including bloodstream infections, liver dysfunction, and metabolic derangements [3,5,16].

The PEPaNIC randomized clinical trial showed that delaying parenteral nutrition for one week in the pediatric ICU proved clinically superior to early initiation, leading to fewer new infections, reduced duration of intensive care dependency, and a shorter overall hospital stay [51]. The sub-analysis of the same study revealed that delaying PN during the first week in these acutely undernourished patients proved clinically superior to early supplementation, resulting in a lower risk of new infections and an increased chance of earlier live discharge. Notably, withholding PN during the initial week was not associated with significant weight deterioration during the PICU stay [52].

Despite growing evidence supporting the superiority of enteral nutrition (EN) in critically ill children, its implementation in clinical practice remains challenging. These challenges often lead to a reliance on PN, with observational studies consistently reporting high rates of PN use, either as the primary mode of nutrition or in combination with minimal trophic feeds [15,16,39]. A study examining predictors of nutrient delivery during the first two weeks of pediatric ECMO therapy found that optimal nutrient delivery was achieved in most patients by day 7, predominantly via PN, while late PN delivery was linked to cumulative protein deficits and failure to meet nutrient delivery goals [16]. The high reliance on PN, especially in the initial phase of ECMO support, warrants further investigation into strategies to optimize EN delivery and minimize the need for PN [11,15].

In terms of caloric and protein delivery, PN offers precise control over macronutrient intake, making it the preferred modality during the early stabilization phase. However, prolonged PN use is associated with hepatic dysfunction, micronutrient imbalances, and suboptimal protein–energy balance, which negatively impact outcomes [6,7,11,44]. Ohman et al. highlighted the variability in caloric delivery across centers and the challenges of achieving nutritional adequacy in patients receiving PN [5].

### 3.10. Challenges in Nutrition Provision

Several factors complicate the provision of adequate nutrition to pediatric ECMO patients. Concerns regarding gut ischemia, often cited as a reason for delayed EN initiation, require careful consideration [2,3]. The use of vasoactive drugs, frequently necessary in ECMO patients, can further complicate nutritional management [3,4].

However, evidence suggests that early EN is generally safe and well-tolerated, even in the presence of vasopressors [8].

Moreover, the need for fluid restriction in some patients can limit the volume of nutrition that can be safely administered [11]. A study by Kerstein et al. found that in a cohort predominantly receiving parenteral nutrition (PN), higher energy and protein adequacy were associated with an increased incidence of hospital-acquired infections (HAIs). This finding highlights a potential drawback of early PN initiation despite achieving nutritional goals within the first five days [11]. The variability in practices across different centers, as highlighted by a multicenter study, underscores the need for standardized protocols and algorithms to guide nutrition support in ECMO patients [5,20]. Furthermore, studies indicate that undernutrition is prevalent among children on ECMO, particularly in those under 2 years of age [7,13], emphasizing the importance of early and aggressive nutrition support [28].

### 3.11. Challenges in Transitioning from Parenteral to Enteral Nutrition

Transitioning from PN to EN in ECMO patients is fraught with challenges, including feeding intolerance, interruptions for procedures, and institutional variability in feeding practices. Greathouse et al. reported that frequent interruptions during diagnostic and therapeutic procedures delay the time to full EN [2]. Multicenter studies, such as those by Ohman et al., have highlighted the need for standardized feeding protocols to reduce variability and ensure adequate caloric delivery [5].

Strategies to optimize the transition include the use of prokinetic agents, adherence to individualized feeding protocols, and monitoring of gastric residual volumes. Irving et al. emphasized that incorporating structured feeding algorithms and multidisciplinary team approach are critical for identifying feeding readiness and managing complications effectively [33].

### 3.12. Impact of Nutritional Strategies on Outcomes

The impact of nutritional strategies on patient outcomes is a crucial area of research in pediatric ECMO. Studies have shown a correlation between adequate nutrition and improved survival rates [6,14]. Early initiation of EN, particularly by day 3 or 5 of ECMO support, is associated with improved survival to hospital [6,14]. Achieving a higher percentage of daily energy intake goals is also protective and linked to better survival [6]. Conversely, larger decreases in weight-for-age z-scores (WAZ) during ECMO are significantly associated with lower survival rates [14]. Moreover, underweight status at admission is an independent predictor of in-hospital mortality [7]. While some studies have shown no significant difference in infection rates between patients receiving EN and those receiving PN, others suggest a potential association between higher nutrient adequacy and increased blood stream infections [11,16]. These findings highlight the complexity of nutritional management in pediatric ECMO and the need for further research to clarify the optimal balance between nutritional adequacy and infection risk. Prospective studies and RCTs are needed to explain how achieving enteral autonomy is linked to improved survival [6,11,14].

### 3.13. Guidelines and Recommendations

Several organizations have published guidelines and recommendations for nutritional support in critically ill pediatric patients, including those on ECMO [10,43,53]. The American Society for Parenteral and Enteral Nutrition (ASPEN) has established specific guidelines for neonates on ECMO, emphasizing the importance of early and aggressive nutrition support with high protein intake (up to 3 g/kg/day) and adequate caloric intake (100–120 kcal/kg/day) [26,28]. The European Society of Pediatric and Neonatal Intensive Care (ESPNIC) has also published position statements and clinical recommendations for nutritional support in critically ill children, emphasizing the importance of early enteral nutrition, particularly in patients on ECMO and other forms of hemodynamic support [43,53].

The ELSO Guidelines for the Provision and Assessment of Nutritional Support in the Neonatal and Pediatric ECMO Patient recommend a baseline nutritional assessment within the first 48 h of admission. Nutritional support should be individualized with frequent evaluations of metabolic needs, fluid balance, and GI tolerance to achieve the best results. The initiation of enteral nutrition is suggested within 48 h of ECMO support or when the patient is clinically stable [49]. A multidisciplinary group of intensivists, ECMO specialists, and dietitians working together is essential to develop nutritional care strategies.

However, there is a lack of large, well-designed randomized controlled trials to provide definitive evidence-based guidelines for this specific population [54]. The variability in practices across different centers and countries underscores the need for further research to establish optimal nutritional practices and assessment tools for pediatric patients on ECMO [3,5,20]. Furthermore, the guidelines emphasize the importance of individualized nutrition plans, considering the patient’s age, underlying condition, and response to treatment [4,10,43,53].

### 3.14. Future Research Opportunities

Despite significant advances, several research gaps remain in the field of pediatric ECMO nutrition. Further research is needed to:

− Develop more accurate methods for assessing energy and protein requirements in this heterogeneous population, considering the influence of underlying conditions, ECMO mode, and CRRT.
− Establish optimal timing and methods for initiating and delivering EN, addressing concerns about gut ischemia and feeding intolerance.
− Investigate the optimal composition of EN formulations, including the use of human milk when indicated versus other formulas and the potential benefits of specialized diets.
− Conduct large, multicenter, randomized controlled trials to compare the effectiveness and safety of different nutritional strategies on long-term outcomes.
− Develop standardized protocols and algorithms for nutritional assessment and support in pediatric ECMO patients to reduce variability in practice and improve outcomes.
− Explore the role of the gut microbiome in response to nutritional interventions and its influence on patient outcomes.
− Investigate the long-term nutritional consequences of ECMO in survivors, particularly those with underlying conditions such as CDH.

## 4. Conclusions

Adequate nutrition is essential for optimizing outcomes in pediatric ECMO patients. While enteral nutrition (EN) is preferred due to its benefits in preserving gut integrity and reducing infection risk, its implementation is often challenging, leading to frequent reliance on parenteral nutrition (PN). Current evidence supports early EN initiation as a strategy to improve survival and reduce morbidity, but further research is needed to refine nutritional approaches and overcome existing barriers.

Standardized assessment tools, feeding protocols, and multicenter collaboration are crucial to enhancing the quality of nutritional care. Developing tailored nutrition support algorithms based on patient age, underlying pathology, and ECMO mode could improve both efficacy and safety. Future research should explore the interplay between nutritional interventions, gut microbiome changes, and long-term outcomes, including growth and development in ECMO survivors.

The decision between EN and PN requires careful consideration of clinical stability, nutritional needs, and institutional practices. While PN remains necessary in critically unstable patients, transitioning to EN should be prioritized whenever feasible to optimize patient outcomes. Standardized feeding protocols and continued research will be key to improving nutritional support and overall care in this vulnerable population.

## Figures and Tables

**Table 1:** Key studies on nutrition provision in pediatric patients on ECMO.

Study Title	First Author et al.	Study Design	Population	Main Results	Conclusion
Observational Studies					
Nutrition Provision in Children with Heart Disease on Extracorporeal Membrane Oxygenation (ECMO)	Kerstein, J.S. et al. (2024) [11]	Retrospective *n* = 252	Neonatal and Pediatric patients with heart disease on ECMO	Median daily caloric intake was 58 kcal/kg/day, and protein intake was 1.4 g/kg/day, both below recommended targets.	Highlighted challenges in providing optimal nutrition and the need for standardized protocols.
Measuring the Resting Energy Expenditure in Children on Extracorporeal Membrane Oxygenation: A Prospective Pilot Study	Ewing, L.J. et al. (2023) [12]	Prospective *n* = 7	Pediatric patients on ECMO	Measured resting energy expenditure was highly variable, ranging from 30 to 120 kcal/kg/day.	Indicated that indirect calorimetry may improve individualized nutrition plans.
Early Enteral Nutrition and Gastrointestinal Complications in Pediatric Patients on Extracorporeal Membrane Oxygenation	Pérez, G. et al. (2022) [13]	Retrospective *n* = 100	Pediatric ECMO patients	60% of patients received enteral nutrition, with 10% experiencing gastrointestinal complications.	Found early enteral feeding feasible with close monitoring.
Vasopressors and Enteral Nutrition in the Survival Rate of Children During Extracorporeal Membrane Oxygenation	Alexander, E. et al. (2022) [14]	Retrospective *n* = 76	Pediatric ECMO patients	76% of patients received enteral nutrition; no difference in mortality based on vasopressor use.	No significant association between vasopressors and feeding intolerance was found.
A Multicenter Study of Nutritional Adequacy in Neonatal and Pediatric Extracorporeal Life Support	Ohman, K. et al. (2020) [5]	Multicenter retrospective *n* = 283	Neonatal and pediatric ECMO patients	61% of energy and 55% of protein targets met by day 7.	Concluded that nutritional delivery was often suboptimal.
Nutritional Practices and Adequacy in Children Supported on Extracorporeal Membrane Oxygenation	Ong, C. et al. (2018) [15]	Retrospective *n* = 51	Pediatric ECMO patients	75% of patients received enteral feeds, with an average energy intake of 55 kcal/kg/day.	Reported frequent undernutrition and suggested improved feeding strategies.
Nutrition Delivery During Pediatric Extracorporeal Membrane Oxygenation Therapy	Armstrong, L.B. et al. (2018) [16]	Retrospective *n* = 54	Pediatric ECMO patients	59% of energy and 46% of protein targets were met.	Found that patients often received inadequate nutrition.
Underweight Status Is an Independent Predictor of In-Hospital Mortality in Pediatric Patients on Extracorporeal Membrane Oxygenation	Anton-Martin, P. et al. (2018) [7]	Retrospective *n* = 491	Pediatric ECMO patients	Underweight patients had a 47% mortality rate vs. 31% in non-underweight patients (*p* = 0.01).	Recommended aggressive nutrition support for underweight patients.
Impact of Early Initiation of Enteral Nutrition on Survival During Pediatric Extracorporeal Membrane Oxygenation	Greathouse, K.C. et al. (2018) [6]	Retrospective *n* = 49	Pediatric ECMO patients	Early enteral nutrition was associated with 18% lower mortality (*p* < 0.05).	Found early enteral feeding associated with improved survival.
Routine Enteral Nutrition in Neonates on Extracorporeal Membrane Oxygenation	Hanekamp, M.N. et al. (2005) [8]	Retrospective *n* = 77	Neonates on ECMO	86% of neonates tolerated enteral feeding without complications.	Found enteral feeding to be safe and beneficial.
The Incidence of Septic Complications in Newborns on Extracorporeal Membrane Oxygenation Is Not Affected by Feeding Route	Wertheim, H.F.L. et al. (2001) [17]	Retrospective *n* = 96	Neonates on ECMO	No significant difference in sepsis rates between enteral and parenteral feeding.	Found no significant difference between enteral and parenteral nutrition.
Introduction of Enteral Feeding in Neonates on Extracorporeal Membrane Oxygenation After Evaluation of Intestinal Permeability Changes	Piena, M. et al. (1998) [18]	Prospective *n* = 9	Neonates on ECMO	Enteral feeding started at median 4 days: no significant increase in intestinal permeability.	Suggested enteral feeding is feasible with careful monitoring.
Total Enteral Nutrition Versus Total Parenteral Nutrition During Pediatric Extracorporeal Membrane Oxygenation	Pettignano, R. et al. (1998) [19]	Retrospective *n* = 29	Pediatric ECMO patients	Enteral feeding led to lower infection rates and shorter ICU stays compared to TPN.	Suggested enteral feeding was beneficial when tolerated.
Surveys					
Nutrition in Neonatal and Pediatric Patients on Extracorporeal Membrane Oxygenation: A Survey of Current Practice in the US and Canada	Furlong-Dillard, J.M. et al. (2024) [20]	Survey	Neonatal and pediatric centers	Only 27% of centers had nutrition guidelines; enteral feeding initiation varied widely.	Suggested the need for standardized nutrition guidelines.
Enteral Nutrition in Neonatal and Pediatric Extracorporeal Life Support: A Survey of Current Practice	Desmarais, T.J. et al. (2015) [21]	Survey	Neonatal and pediatric ECMO centers	56% of centers used enteral nutrition routinely; timing and strategies varied.	Emphasized the need for consistent nutrition protocols.

**Table 2:** Potential causes of enteral feeding intolerance and mitigation strategies in critically ill children.

Category	Potential Causes and Signs [45,46,47,48]	Management Strategies [10,26,46,47,48,49]
Gastric	Causes: - Delayed gastric emptying (gastroparesis) - Gastroesophageal reflux Signs: - Vomiting - High gastric residual volumes (GRVs)	- Initiate prokinetic therapy (e.g., erythromycin, metoclopramide) as indicated - Minimize sedative and neuromuscular blockade use - Maintain head-of-bed elevation (30–45°) - Consider post-pyloric feeding in cases of persistent high GRVs - Low volume bolus feeds vs. continuous feeds
Small Intestine	Causes: - Impaired motility (intestinal ileus) - Bowel ischemia - Malabsorption syndromes (e.g., short bowel syndrome, pancreatic insufficiency) - Small intestinal bacterial overgrowth (SIBO) Signs: - Abdominal distention - Increased intra-abdominal pressure (IAP) - Rising lactate level - Absent bowel output	- Measure IAP - Consider trophic enteral nutrition to promote gut motility - Optimize hemodynamic support and avoid overfeeding - Utilize elemental or semi-elemental formulas in malabsorption states - Consider probiotics in select patient populations - Laxatives and prokinetics - Change enteral formula when applicable
Large Intestine	Causes: - Dysbiosis and gut microbiome alterations - Bowel ischemia Signs: - Diarrhea (osmotic or secretory) - Constipation and fecal impaction	- Adjust formula osmolality and reduce excessive carbohydrate loads - Ensure adequate hydration and fiber intake - Implement pharmacologic interventions (e.g., stool softeners, laxatives) as needed - Consider probiotic or prebiotic supplementation to support gut microbiota balance

## Abbreviations

The following abbreviations are used in this manuscript:

EN	Enteral nutrition
PN	Parenteral nutrition
ECMO	Extracorporeal membrane oxygenation
ASPEN	American Society for Parenteral and Enteral Nutrition
ESPEN	European Society of Clinical Nutrition and Metabolism
PICU	Pediatric intensive care units
ELSO	Extracorporeal Life Support Organization
ECPR	Extracorporeal cardiopulmonary resuscitation
VA	Veno-arterial
VV	Veno-venous
